# Supplementary material for: Therapeutic Interaction of Apatinib and Chidamide in T-cell Acute Lymphoblastic Leukemia through Interference with Mitochondria Associated Biogenesis and Intrinsic Apoptosis
Source: J Pers Med. 2021 Sep 29;11(10):977. doi: 10.3390/jpm11100977 (PMC8540063; doi:10.3390/jpm11100977)
Supplement: Supplementary file 1 [file jpm-11-00977-s001.zip › jpm-1381161-supplementary.pdf]

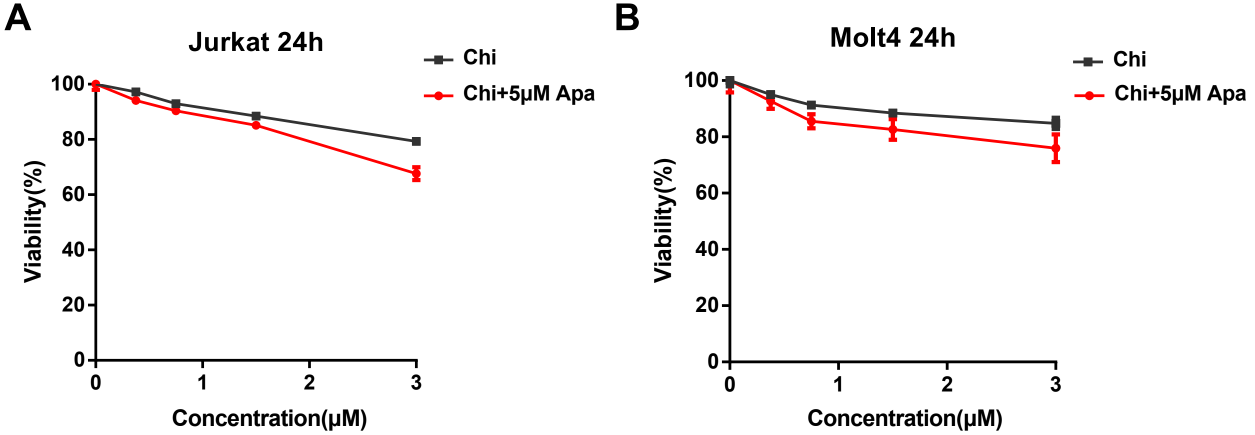
A
Jurkat 24h
Chi
Chi+5μM Apa
Viability(%)
0
20
40
60
80
100
0
1
2
3
Concentration(μM)
B
Molt4 24h
Chi
Chi+5μM Apa
Viability(%)
0
20
40
60
80
100
0
1
2
3
Concentration(μM)

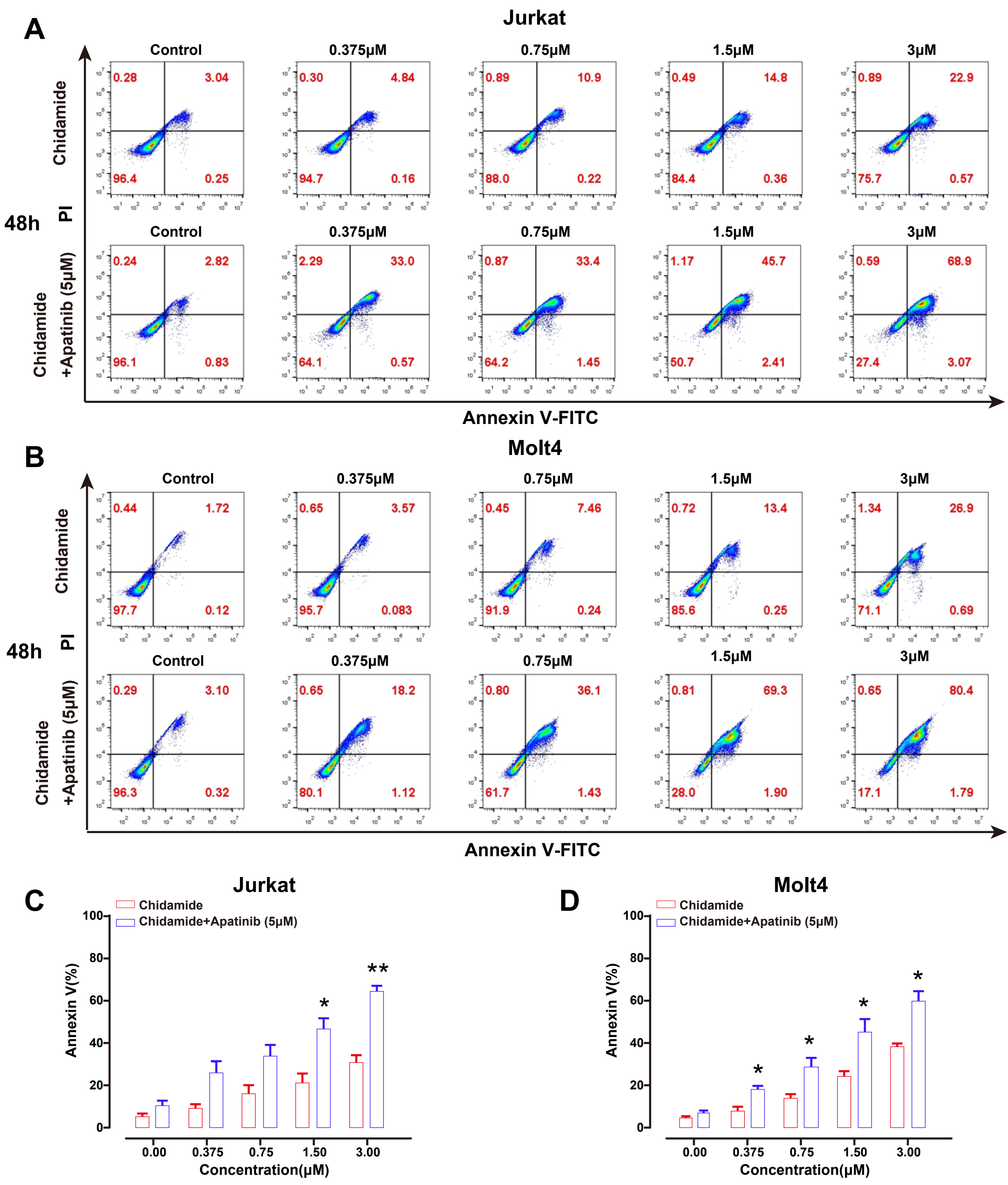

A
Jurkat
48h
Chidamide
Chidamide +Apatinib (5μM)
PI
Control
0.375μM
0.75μM
1.5μM
3μM
0.28
3.04
96.4
0.25
0.30
4.84
94.7
0.16
0.89
10.9
88.0
0.22
0.49
14.8
84.4
0.36
0.89
22.9
75.7
0.57
0.24
2.82
96.1
0.83
2.29
33.0
64.1
0.57
0.87
33.4
64.2
1.45
1.17
45.7
50.7
2.41
0.59
68.9
27.4
3.07
Annexin V-FITC
B
Molt4
48h
Chidamide
Chidamide +Apatinib (5μM)
PI
Control
0.375μM
0.75μM
1.5μM
3μM
0.44
1.72
97.7
0.12
0.65
3.57
95.7
0.083
0.45
7.46
91.9
0.24
0.72
13.4
85.6
0.25
1.34
26.9
71.1
0.69
0.29
3.10
96.3
0.32
0.65
18.2
80.1
1.12
0.80
36.1
61.7
1.43
0.81
69.3
28.0
1.90
0.65
80.4
17.1
1.79
Annexin V-FITC
C
Jurkat
Chidamide
Chidamide+Apatinib (5μM)
Annexin V(%)
0
20
40
60
80
100
0.00
0.375
0.75
1.50
3.00
Concentration(μM)
*
**
D
Molt4
Chidamide
Chidamide+Apatinib (5μM)
Annexin V(%)
0
20
40
60
80
100
0.00
0.375
0.75
1.50
3.00
Concentration(μM)
*
*
*
*

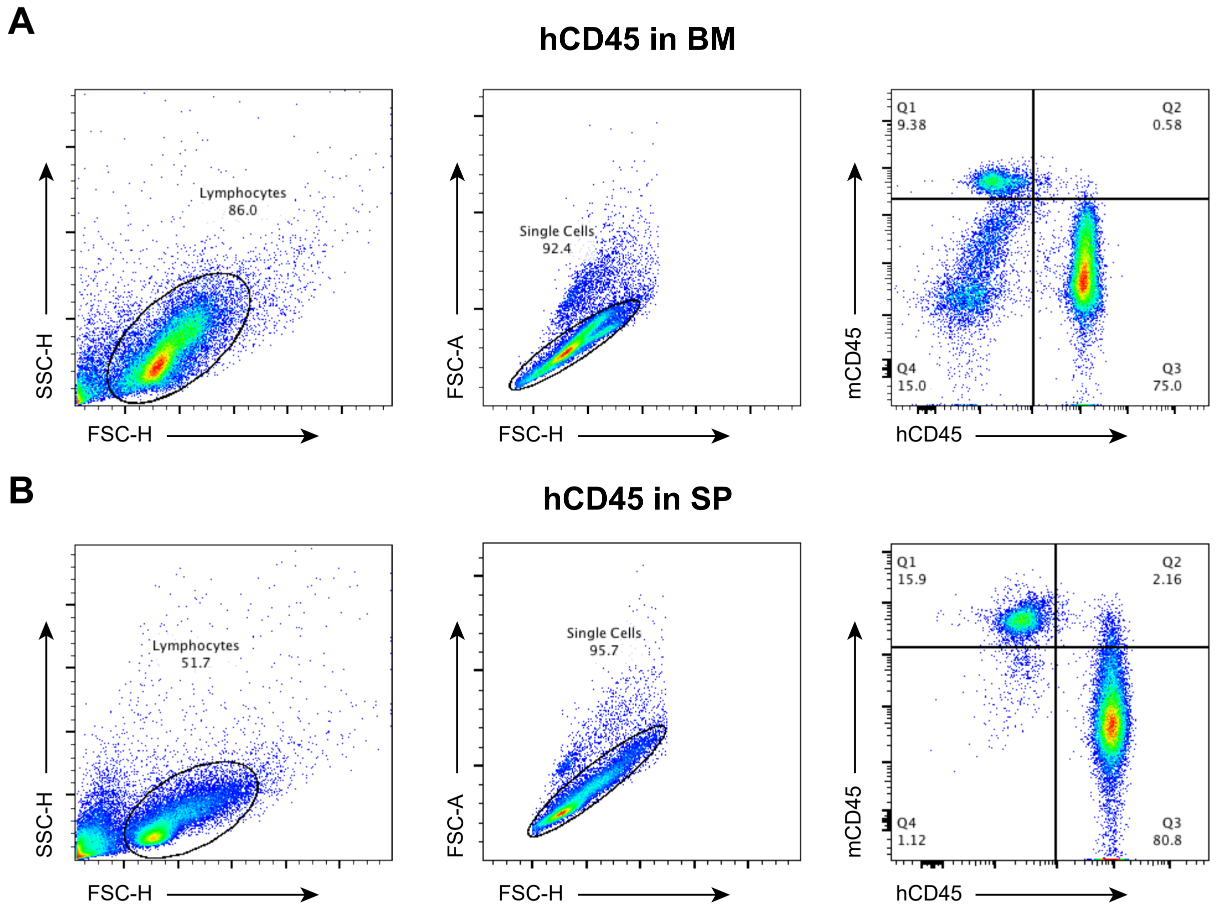
A
hCD45 in BM
Lymphocytes
86.0
SSC-H
FSC-H
Single Cells
92.4
FSC-A
FSC-H
Q1
9.38
Q2
0.58
Q4
15.0
Q3
75.0
mCD45
hCD45
B
hCD45 in SP
Lymphocytes
51.7
SSC-H
FSC-H
Single Cells
95.7
FSC-A
FSC-H
Q1
15.9
Q2
2.16
Q4
1.12
Q3
80.8
mCD45
hCD45

Supplementary Table S1. Primer sequences for qRT-PCR assay.

**qRT-PCR primer sequence**		
Primers	Forward/Reverse	5'-3'
β-actin	F	AGCGAGCATCCCCCAAAGTT
	R	GGGCACGAAGGCTCATCATT
COX10	F	AGGTTGCGTAGGAGGCTCTGTC
	R	GCTGTGTGACATACATGCGTTTGAG
MPC1	F	CGAAGCAAGGATTTCCGGGACTAC
	R	TTGATGGCAGCAATGGGAAGACC
SDHD	F	CTTGTCACCGAGCCACCATTCTG
	R	GGAATAGTCCATCGCAGAGCAAGG
MDH1	F	CGTCAGGGACATCTGGTTTGGAAC
	R	GGAACACCATAGGAGTTGCCATCAG
PDHA1	F	CACAGACCATCTCATCACAGCCTAC
	R	CCTCCTTTCCCTTTAGCACAACCTC
CS	F	TGCCCTTTCCGACCCTTACCTG
	R	TGTGTTAGCCAGACAAGCACTTCC

Supplementary Table 2. Clinical characteristics of a T-ALL patient whose blast cells were employed to establish PDX model.

Disease Characteristics	Specific information
Phenotype	T-ALL
Gender	Male
Age (years)	31
WBC	289.44*10^9/L
HGB	129 g/L
PLT	11*10^9/L
LDH	2091 U/L
Percentage of blast cells in bone marrow	92.5%
Percentage of blast cells in peripheral blood	92%
Karyotype	TCRB, TCRG, TCRD rearrangement positive
Genetic alterations	NOTCH1 nonsense mutation, PTEN frameshift mutation
Xenograft engraftment method	Tail vein injection
